# Prevalence and management of eosinophilia based on periodic health examinations in primary care clinics

**DOI:** 10.2478/abm-2022-0030

**Published:** 2023-06-16

**Authors:** Thareerat Ananchaisarp, Panya Chamroonkiadtikun, Jakrawadee Julamanee, Kewalee Perdvong, Thitawan Chimpalee, Nutnicha Rattanavirakul, Nattawat Leelarujijaroen, Tiprada Hathaipitak, Thanarat Tantinam

**Affiliations:** Division of Family and Preventive Medicine, Faculty of Medicine, Prince of Songkla University, Songkhla 90110, Thailand; Division of Internal Medicine, Faculty of Medicine, Prince of Songkla University, Songkhla 90110, Thailand; Faculty of Medicine, Prince of Songkla University, Songkhla 90110, Thailand

**Keywords:** anthelmintic, eosinophilia, prevalence, therapeutics, periodic health examinations

## Abstract

**Background:**

Eosinophilia is a common, hematologic abnormality detected in periodic health checkups with diverse etiologies. There are a few clinical practice guidelines for the management of eosinophilia.

**Objectives:**

To determine the prevalence of eosinophilia among patients undergoing periodic health examinations, evaluate its management and outcomes, and identify its associated factors.

**Methods:**

We conducted a retrospective study that included patients with eosinophilia diagnosed during the 2018 periodic health examinations at Songklanagarind Hospital.

**Results:**

The prevalence rate of eosinophilia was 9.6% (988/10,299), and most patients (52.6%) were male with a median age of 53.0 (42.0–61.0) years. Only 174 patients (17.6%) were diagnosed and further examined to identify the cause of eosinophilia; including an examination of medical history (18.4%), physical examination (93.1%), laboratory analysis (9.2%), and consultation with internists (14.9%). Empirical anthelmintic therapy was administered in 130 patients (74.7%), and 49.2% achieved resolution. The possible causes of eosinophilia were identified in 20.7% (204/988), the most common cause being atopic disease (51.5%). Patients with moderate-to-severe eosinophilia were significantly more likely to be diagnosed, undergo further laboratory tests, and proceed with consultations with internists (adjusted OR [95% CI] = 3.52 [1.97–6.32], 17.13 [5.74–51.11], and 6.38 [1.95–20.93], respectively).

**Conclusions:**

Eosinophilia is commonly identified in periodic health examinations, and most primary physicians lack knowledge regarding the diagnostic work-up required to determine the cause of eosinophilia. Empirical anthelmintic therapy showed satisfactory efficacy for the management of eosinophilia in areas where parasite infection is endemic.

Eosinophilia is a condition in which the eosinophil count in the peripheral blood exceeds the normal range; there are many ways to define eosinophilia, but the most popular adhered- to definition is an absolute eosinophil count (AEC) of >500 cells/μL [1,2,3,4,5]. Depending on the severity, eosinophilia can be classified into mild (AEC 501–1499 cells/μL), moderate (AEC 1500–4999 cells/μL), and severe (AEC ≥5000 cells/μL) [2, 4,5,6,7]. Eosinophilia is a common condition often diagnosed based on the results of an automated complete blood count (CBC); its prevalence ranges from 0.1% to 77.5% [6,7,8,9,10] depending on the study setting and population. The underlying causes of eosinophilia can be broadly divided into 3 categories as follows [1, 2, 9, 11, 12]: (1) secondary/reactive eosinophilia, which results from cytokine-driven reactive phenomena from multiple primary conditions, such as atopy and parasite infection, and is the most common cause of eosinophilia; (2) clonal eosinophilia in which mutation causes the clonal expansion of eosinophils, for example in hematological neoplasms; and (3) idiopathic eosinophilia in which the cause cannot be identified even after thorough investigation.

According to the guideline for the investigation and management of eosinophilia [1, 11], eosinophilia evaluation should include a complete medical history and physical examination, especially for symptoms and signs of malignancy, such as fever, anemia, lymphadenopathy, or hepatosplenomegaly. Regarding laboratory investigations, patients with mild eosinophilia and no warning signs should be investigated only for the suspected cause, which is most commonly reactive eosinophilia. Meanwhile, moderate-to-severe eosinophilia could be associated with serious conditions, such as hematologic malignancies [10, 12,13,14]; thus, these patients require further investigation to identify the underlying cause and assess organ damage [2, 15, 16].

A previous study reported that 40.0% of ambulatory physicians in Canada did not interpret the eosinophil count in CBC results and did not request further diagnostic work-up for eosinophilia [6]. In another study of returning travelers in Israel [17], 47.5% of eosinophilia patients underwent invasive investigation; however, the underlying cause of eosinophilia was not identified in most cases (n = 16, 88.9%). Moreover, in the aforementioned study, most eosinophilia patients were treated with empirical anthelmintic drugs, and 85.7% experienced eosinophilia resolution.

In Thailand, there are no clinical practice guidelines for the management of eosinophilia; thus, patient management largely depends on the physician’s attention, the successful diagnosis of eosinophilia, and subsequent work-ups following diagnosis. Moreover, there are only a few studies on the prevalence and physician management of eosinophilia in Thailand, especially during periodic health checkups where most eosinophilia patients are asymptomatic. Thus, this study aimed to determine the prevalence of eosinophilia and identify patterns of physician management and patient outcomes among eosinophilia patients receiving periodic health examinations at the Songklanagarind Hospital.

## Materials and methods

The study protocol was approved by the Office of the Human Research Ethics Committee of the Prince of Songkla University (Certificate of approval No. 64-304-9-1). The requirement for informed consent was waived owing to the retrospective nature of the study.

### Study design

A retrospective study was conducted between January 1, 2018 and December 31, 2018, with a 3-year follow-up period. The study setting consisted of a primary care unit (PCU), general practice (GP), and premium checkup (a private clinic serviced by a specialist physician) clinics that provide health checkup services in Songklanagarind Hospital, a tertiary care hospital with a medical school, residency training programs, and a referral center in the South of Thailand.

### Study sample and sampling

All patients aged ≥15 years who underwent a CBC test for periodic health examinations, including both healthy individuals undergoing routine health checkups and patients with chronic diseases undergoing annual tests, in the PCU, GP, or premium checkup clinics at Songklanagarind Hospital in 2018 were enrolled; we aimed to evaluate the prevalence of eosinophilia among these patients. Eosinophilia was defined as an AEC of >500 cells/μL. We enrolled all eosinophilia patients to determine patterns of patient management, which was the secondary objective of this study. The sample size was calculated by estimating the population proportion formula, that is, n = [(Z_1–α/2_)^2^p(1–p)]/d^2^ = 372; the proportion of patients with eosinophilia who received further investigation in Canada was 0.59 [6] and Error (d) = 0.05.

### Variables

The dependent variables consisted of diagnosis, management, and outcome of eosinophilia patients. An eosinophilia diagnosis was defined when the physician noted the term “eosinophilia” in the medical records, when a diagnosis was made based on the ICD-10 system, or when both of these conditions were satisfied. The diagnostic work-up for eosinophilia included the collection of complete medical history, physical examination, laboratory tests, and consultation with an internist. The physician’s diagnosis of eosinophilia was used to indicate their intention to investigate the underlying cause of eosinophilia with further work-up. The outcomes of eosinophilia patients were assessed by searching a possible cause of eosinophilia in every medical record until October 31, 2021, with a follow-up duration of 3 years. Due to the lack of previous studies investigating the factors associated with the management and outcome of eosinophilia patients, we used sex, age, disease severity, and CBC parameter as independent variables.

### Data collection

The CBC results of all eligible patients were collected, and AEC was calculated by multiplying the white blood cell count with the percentage of eosinophils. Patients with an AEC of >500 cells/μL were selected and reviewed for relevant information regarding eosinophilia management and outcomes using the hospital information system. Data were recorded in the case record form. All researchers were trained to precisely understand the meaning of each question before commencement of data collection.

### Data management and analysis

Data were logged in EpiData software (version 3.1, EpiData Association) on a double entry basis and analyzed using R (R Core Team 2021). Descriptive analysis was used to analyze the patients’ baseline characteristics, patient management, and outcomes. Categorical data are presented as number (percentage), and continuous data are presented as the median (Q1, Q3) when normal distribution assumptions were not met. Factors associated with physician’s diagnosis and diagnostic work-up for eosinophilia were analyzed using multiple logistic regression analysis models; we included all independent variables. Statistical significance was set at *P* < 0.05 using a 2-tailed test.

## Results

A total of 10,299 patients underwent CBC tests for periodic health examinations at the PCU, GP, and premium checkup clinics in 2018. Their baseline characteristics stratified by eosinophilia status are shown in **Table 1**. Of these, 988 patients had eosinophilia (9.6%), with a male-to-female ratio of 1.10 and a median age (Q1–Q3) of 53.0 (42.0–61.0) years. The prevalence of eosinophilia in each clinic was proportional to the number of CBC requests, with the highest prevalence in the GP clinic, followed by the premium checkup and PCU clinics. When classified for severity of eosinophilia, most patients had mild eosinophilia (91.9%), 76 patients had moderate eosinophilia (7.7%), and only 4 patients had severe eosinophilia (0.4%). In the group of eosinophilia patients, only 174 patients (17.61%) were diagnosed and received management from a primary doctor. Compared against patients who did not receive management, those who had received it had significantly higher AECs. Moreover, among patients who had received management, a significant variation from the reminder was observed based on the type of clinic that analyzed the CBC results.

**Table 1.** Baseline characteristics of the study participants (n = 10,299).

**Characteristics**	**Without eosinophilia (n = 9311)**	**With eosinophilia (n = 988)**		

		**Having management (n = 174)**	**No management (n = 814)**	***P***
Sex, n (%)				0.625†
Male	6287 (67.5)	95 (54.6)	425 (52.2)	
Female	3024 (32.5)	79 (45.4)	389 (47.8)	
Age, years, median (Q1, Q3)	51.0 (40.0, 60.0)	53.0 (41.2, 61.0)	53.0 (43.0, 61.0)	0.676‡
Clinic that analyzed CBC results, n (%)				<0.001†
GP	6927 (74.4)	73 (42.0)	684 (84.0)	
Premium checkup clinic	1978 (21.2)	97 (55.7)	100 (12.3)	
Primary care unit	406 (4.4)	4 (2.3)	30 (3.7)	
AEC, cells/uL, median (Q1, Q3)	152.6 (88.5, 250.0)	811.7 (602.5, 1178.0)	666.4 (567.5, 926.9)	<0.001‡

† Chi-square tests.

‡ Ranksum test.

AEC, absolute eosinophil count; CBC, complete blood count; GP, general practice.

A total of 174 eosinophilia patients received a diagnosis and management (**Table 2**). Of these, 18.4% were asked questions relating to their health history to determine the etiology of eosinophilia, including atopy and risk of parasite infestation. Most of them had noted at least 1 physical examination that can help to identify the underlying cause of eosinophilia; the common abnormal examination was abnormal lung sound (90.8%), followed by hepatosplenomegaly (62.6%). Approximately 13.0% of physicians noted an abdominal mass or lymphadenopathy. A laboratory investigation to ascertain the work-up cause of eosinophilia was requested only in the case of 16 eosinophilia patients at the first clinic, and most of these patients were requested to undergo a stool examination. Approximately 75.9% of them received an anthelmintic drug; the most commonly prescribed drug was albendazole for 1–7 d (n = 123), and some physicians added ivermectin. Almost all patients who received an anthelmintic drug were prescribed an empirical regimen (125 patients did not undergo the stool examination and 5 patients received an anthelmintic drug although the stool microscopy returned a normal result), and approximately 49.2% of them showed resolution. Approximately 14.9% of eosinophilia patients were referred to internists for consultation about eosinophilia.

**Table 2.** Type of management received by patients with eosinophilia classified by clinic that analyzed CBC results (n = 174).

**Type of management**	**GP (n = 73)**	**Primary care unit (n = 4)**	**Premium checkup (n = 97)**	**Total (n = 174)**
History	20 (27.4)	0	12 (12.4)	32 (18.4)
Atopic history	14 (19.2)	0	10 (10.3)	24 (13.8)
Risk of parasite infection†	10 (13.7)	0	2 (2.1)	12 (6.9)
Photosensitive rash, polyarthritis	1 (1.4)	0	0	1 (0.6)
Family history of eosinophilia	0	0	0	0
Physical examination	63 (86.3)	2 (50.0)	97 (100.0)	162 (93.1)
Abnormal lung sound	60 (82.2)	2 (50.0)	96 (99.0)	158 (90.8)
Skin lesion	21 (28.8)	1 (25.0)	14 (14.4)	36 (20.7)
Hepatosplenomegaly	18 (24.7)	0	91 (93.8)	109 (62.6)
Abdominal mass	18 (24.7)	0	5 (5.2)	23 (13.2)
Lymphadenopathy	4 (5.5)	0	17 (17.5)	21 (12.1)
Laboratory investigation	13 (17.8)	0	3 (3.1)	16 (9.2)
Repeat CBC	2 (2.7)	0	0	2 (1.1)
Stool examination	11 (15.1)	0	3 (3.1)	14 (8.0)
Others‡	0	0	0	0
Anthelmintic drug was prescribed	51 (69.9)	4 (100.0)	77 (79.4)	132 (75.9)
No request for stool examination	44 (60.3)	4 (100.0)	77 (79.4)	125 (71.8)
Stool examination returning a normal result	5 (6.8)	0	0	5 (2.9)
Stool examination returning an abnormal result	2 (2.7)	0	0	2 (1.1)
Internist consultation	10 (13.7)	0	16 (16.5)	26 (14.9)

Data are presented as number (%).

† From eating raw cooked food, walking barefoot, or traveling to an endemic area prone to parasite infections.

‡ Others included serology for infection (hepatitis B and C virus, human immunodeficiency virus, parasite), autoantibody, skin prick test, and imaging studies (ultrasound, computed tomography, magnetic resonance imaging).

CBC, complete blood count; GP, general practice.

**Figure 1.**
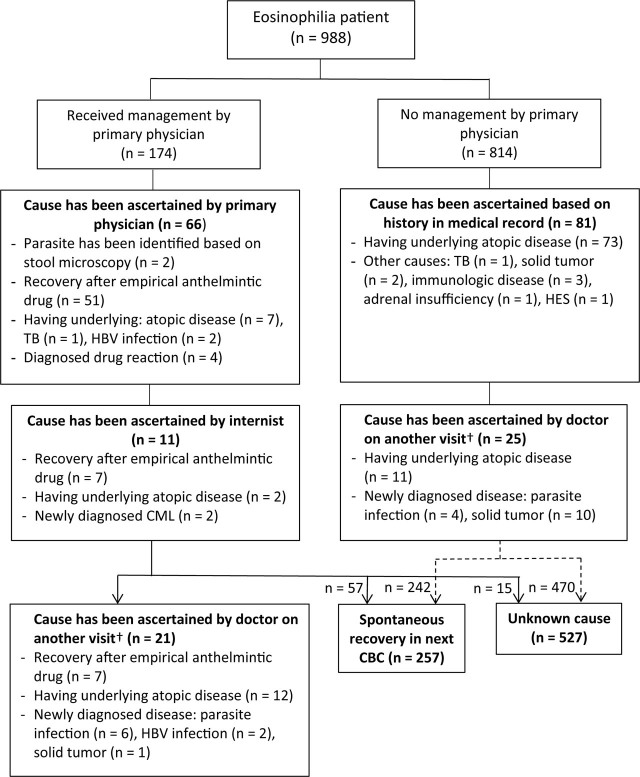
Details of management and outcome of eosinophilia (n = 988). †Based on review of medical records, diseases were diagnosed within 3 years after the visit where CBC tests showed eosinophilia. CBC, complete blood count; CML, chronic myeloid leukemia; HBV, hepatitis B virus; HES, hypereosinophilic syndrome; TB, tuberculosis.

The details of flow management and outcome data for 988 eosinophilia patients are shown in **Figure 1**. The primary physician identified the possible cause of eosinophilia in 37.9% of diagnosed patients, and the internists confirmed the cause of eosinophilia in 11 cases. Approximately 4.7% of eosinophilia patients (n = 46) were noted diseases which was possible cause of eosinophilia in OPD note by another physician in later hospital visit. Approximately 26.0% of eosinophilia patients (n = 257) showed a spontaneous recovery in the next CBC results, of which only 3 CBC tests were ordered for follow-up. The cause of eosinophilia was unknown in 53.3% of the eosinophilia patients. Of these, 14.4% had persistent eosinophilia without an identified cause, despite undergoing further investigation by a primary physician or internist.

Among 988 eosinophilia patients, we could identify the possible cause of eosinophilia in 204 cases (20.7%). The details of the possible causes of eosinophilia are shown in **Table 3**; approximately 44.6% of them had underlying diseases associated with eosinophilia. The most common cause of eosinophilia was atopic disease (51.5%), of which 80.0% was constituted by cases of previously underlying disease. The second most common cause was infection (36.8%); most of these cases were characterized by parasite infestation, with about three-quarters diagnosed by a primary physician. Thirteen patients (6.4%) had a solid tumor, and most of these cases were detected by other doctors during another visit. Additionally, an internist diagnosed 2 patients with CML during the diagnostic work-up for eosinophilia.

**Table 3.** Prevalence of possible causes of eosinophilia stratified by diagnosis (n = 204).

**Possible causes**	**Total**	**Known underlying disease (n = 91)**	**Diagnosed by the primary physician (n = 66)**	**Diagnosed by an internist (n = 11)**	**Diagnosed by a doctor on another visit† (n = 36)**
Atopic disease	105 (51.5)	84 (80.0)	7 (6.7)	2 (1.9)	12 (11.4)
Allergic rhinitis	65 (31.9)	56 (86.2)	2 (3.1)	0	7 (10.7)
Asthma	23 (11.3)	20 (87.0)	0	1 (4.3)	2 (8.7)
Allergic rhinitis and asthma	7 (3.4)	7 (100.0)	0	0	0
Atopic dermatitis	3 (1.5)	1 (33.3)	1 (33.3)	0	1 (33.3)
Urticaria	4 (2.0)	0	2 (50.0)	0	2 (50.0)
Food allergy	2 (1.0)	0	1 (50.0)	1 (50.0)	0
Allergic conjunctivitis	1 (0.5)	0	1 (100.0)	0	0
Infection	75 (36.8)	1 (1.3)	55 (73.4)	7 (9.3)	12 (16.0)
Parasite infestation	70 (34.3)	0	53 (75.7)	7 (10.0)	10 (14.3)
Viral infection	3 (1.5)	0	1 (33.3)	0	2‡ (66.7)
TB	2 (1.0)	1 (50.0)	1 (50.0)	0	0
Solid tumor	13 (6.4)	2 (15.4)	0	0	11 (84.6)
Breast cancer	5 (2.5)	1 (20.0)	0	0	4 (80.0)
Prostate cancer	3 (1.5)	1 (33.3)	0	0	2 (66.7)
Lung cancer	2 (1.0)	0	0	0	2 (100.0)
Rectal cancer	1 (0.5)	0	0	0	1 (100.0)
Ovarian cancer	1 (0.5)	0	0	0	1 (100.0)
Parotid cancer	1 (0.5)	0	0	0	1 (100.0)
Drug reaction: allopurinol	4 (2.0)	0	4 (100.0)	0	0
Autoimmune disease	3 (1.4)	3 (100.0)	0	0	0
Rheumatoid arthritis	1 (0.5)	1 (100.0)	0	0	0
Psoriasis	1 (0.5)	1 (100.0)	0	0	0
Autoimmune thyroid disease	1 (0.5)	1 (100.0)	0	0	0
Hematologic disease	3 (1.4)	1 (33.3)	0	2 (64.7)	0
HES	1 (0.5)	1 (100.0)	0	0	0
Chronic myeloid leukemia	2 (1.0)	0	0	2 (100.0)	0
Adrenal insufficient	1 (0.5)	0	0	0	1 (100.0)

Data are presented as number (%).

† Based on review of medical records, diseases were diagnosed within 3 years after the visit where CBC tests showed eosinophilia.

‡ Hepatitis B infection.

CBC, complete blood count; HES, hypereosinophilic syndrome; TB, tuberculosis.

The factors associated with diagnosis, diagnostic work-up, internist consultation, and identification of the cause of eosinophilia are shown in **Table 4**. Patients examined in the premium checkup clinic were significantly more likely to be diagnosed with eosinophilia than those in the GP clinic (adjusted odds ratio [OR] (95% confidence interval [CI]) = 4.59 [1.57–13.42]). Patients with moderate-to-severe eosinophilia were significantly more likely to be diagnosed, undergo further laboratory tests, and consult with an internist than those with mild eosinophilia (adjusted OR [95% CI] = 3.29 [1.82–5.95], 4.67 [5.37–48.14], and 4.67 [1.38, 19.94], respectively).

**Table 4.** Factors associated with the primary physician’s management and identified cause of eosinophilia (n = 988).

**Factor**	**Diagnosis of eosinophilia**		**Sending laboratory diagnostic work-up of eosinophilia**		**Internist consultation**		**Identified cause of eosinophilia**	

	**Adjusted OR (95% CI)**	***P***	**Adjusted OR (95% CI)**	***P***	**Adjusted OR (95% CI)**	***P***	**Adjusted OR (95% CI)**	***P***
Age ≥60 years (n = 289)	0.93 (0.62, 1.41)	0.747	0.40 (0.11, 1.44)	0.163	1.15 (0.40, 3.25)	0.798	0.91 (0.57, 1.46)	0.704
Sex: Female (n = 468)	0.91 (0.63, 1.33)	0.625	0.58 (0.19, 1.78)	0.339	0.78 (0.30, 2.07)	0.625	1.29 (0.84, 1.96)	0.243
Clinic								
GP (n = 757)	1		1		1		1	
PCU (n = 34)	0.82 (0.19, 3.55)	0.789	0 (0, Inf)	0.995	0 (0, Inf)	0.993	0.42 (0.06, 2.75)	0.367
Premium checkup (n = 197)	4.59 (1.57, 13.42)	0.005*	0.33 (0, 26.89)	0.619	2.06 (0.06, 67.49)	0.685	0.88 (0.21, 3.73)	0.860
Physician								
Intern (n = 765)	1		1		1		1	
Resident FM (n = 11)	0 (0, Inf)	0.975	0 (0, Inf)	0.997	0 (0, Inf)	0.996	1.76 (0.17, 18.62)	0.640
Staff FM (n = 212)	2.39 (0.82, 6.98)	0.110	2.76 (0.03, 227.88)	0.652	6.01 (0.18, 199.46)	0.315	1.40 (0.33, 5.91)	0.646
CBC results								
WBC ≥10,000 cells/μL (n = 104)	0.85 (0.47, 1.54)	0.593	0 (0, Inf)	0.991	1.54 (0.44, 5.31)	0.497	0.51 (0.22, 1.15)	0.105
Severity of eosinophilia								
Mild (n = 908)	1		1		1		1	
Moderate to severe (n = 80)	3.52 (1.97, 6.32)	<0.001*	17.13 (5.74, 51.11)	<0.001*	6.38 (1.95, 20.93)	0.002 *	1.94 (0.98, 3.83)	0.056
Anemia† (n = 102)	1.63 (0.91, 2.94)	0.102	1.79 (0.43, 7.40)	0.422	1.78 (0.48, 6.63)	0.392	0.65 (0.30, 1.40)	0.271
Abnormal platelet count‡ (n = 27)	1.11 (0.40, 3.11)	0.840	3.35 (0.58, 19.44)	0.178	0.76 (0.08, 7.19)	0.810	1.005 (0.29, 3.47)	0.994

† Anemia is defined as a hemoglobin of <13.0 mg/dL in males and <12.0 mg/dL in females.

‡ Abnormal platelet count is defined as a platelet count of <150,000 cells/μL or >450,000 cells/μL.

* *P*< 0.05.

CBC, complete blood count; CI, confidence interval; FM, family medicine; GP, general practice; OR, odds ratio; PCU, primary care unit; WBC, white blood cell count.

## Discussion

We found that the prevalence of eosinophilia among patients receiving periodic health examination in Songklanagarind Hospital was lower than that reported in Northeastern Thailand, including the rural area of Maha Sarakham (14.0%) [18] and Sakonnakorn Hospital (18.6%) [7]. This may be due to the lower prevalence of parasite infection, which constitutes the most common cause of eosinophilia in developing countries [19], in Southern Thailand in comparison with Northeastern Thailand [20]. Moreover, the prevalence rate in this study was lower than that in a previous study in the rural area of Tak (77.5%) [8], where people have limited access to the health care system and there is a high prevalence of parasite infections. Contrastingly, our reported prevalence was higher than that of a study in Denmark (4.0%) [5]. Developed countries usually have low parasite infection rates.

The baseline characteristics of the patients in our study were similar to those of patients in previous studies conducted in Maha Sarakham and Canada [6, 8]. Most patients in this study had mild severity, similar to that reported in previous studies [2, 7, 10, 12, 13]. We found that most eosinophilia patients were not diagnosed and did not receive any management, indicating lack of knowledge or awareness regarding eosinophilia among physicians in Thailand compared with those in Canada and Israel [6, 17]. Against the backdrop of the recommendation of the current clinical practice guidelines, as suggested by Butt et al. [1] and Guo and Bochner [11], it would be appropriate to say that most patients in the present study received inadequate diagnostic work-up that would enable the identification of eosinophilia as the cause of the reported symptoms. Only a few eosinophilia patients were investigated regarding their history associated with the cause of eosinophilia. Most investigations were usually conducted for atopic disease and risk of parasite infection, reflecting the differential diagnosis of the most common cause of eosinophilia in tropical developing countries, including Thailand [14, 16, 20]. Our results demonstrated that most patients diagnosed with eosinophilia had documented physical examinations, which are useful for defining disease etiology. However, the notion that the physical examinations were performed solely for the management of eosinophilia may be an over-interpretation because of the possibility that such physical examination can be a routine part of periodic health examinations. Only a few physicians who diagnosed eosinophilia had documented lymphadenopathy and abdominal mass, which are signs of underlying malignancies [10]. Only a few patients underwent further investigation to identify the cause of eosinophilia. A common investigation in Thailand is stool examination. Requests for intensive investigations, such as imaging studies or skin prick tests, which were performed in a previous study in Canada [6], were not made. This might be due to the high prevalence of parasite infections and the limited health care resources in Thailand. Stool examination is useful in identifying the types of parasite infection, which inform the use of appropriate anthelmintic regimens. However, multiple stool tests to increase sensitivity [6, 17, 21,22,23] may be inconvenient. This may be the reason why one-third of the primary physicians prescribed empirical anthelmintic drugs, a fraction that is the same as the one observed in multiple previous studies [1, 21,22,23,24]. The most popular drug prescribed was albendazole, which is efficient for both intestinal and tissue-invading nematode infections (the cure rate was reported as 27.2%–100%) [25]. Only a few physicians added ivermectin to increase the cure rate for Strongyloidosis [26].

Regarding the results of investigations in eosinophilia patients, the condition most associated with eosinophilia was atopic diseases, consistent with the leading cause mentioned in the Butt et al.’s guideline [1] and the previously reported causes in developed countries [6, 18, 19, 27]. The prevalence of parasite infection, the second most common cause of eosinophilia, is similar to a previous study in another university hospital in Thailand [28] wherein empirical anthelmintic treatments were given. The resolutions of eosinophilia after empirical anthelmintic treatment were used as evidence in support of the assumption that parasite infection is the cause of eosinophilia. Therefore, in areas characterized by a high prevalence of parasite infestation, the prescription of empirical anthelmintic drugs is commonly opted for in the case of individuals with eosinophilia. In most instances, these strategies have shown satisfactory outcomes without serious adverse events [29]. Approximately a quarter of the patients spontaneously recovered from eosinophilia in the next CBC without evidence of any treatment. This can be explained by possible self-remission conditions, such as some viral infections or the remission phase of atopic diseases [18]. Additionally, some patients might receive anthelmintic drugs from other sources, such as over-the-counter.

The severity of eosinophilia was significantly associated with diagnoses, laboratory investigations, and internist consultations in the quest for the causations and potential interventions. These practices are consistent with recent clinical practice guidelines [1, 11]. We suggest that all physicians should pay attention to the need for ascertaining the specific cause of eosinophilia, and accordingly request for diagnostic work-up for the cause to be ascertained, especially when the condition is moderate to severe. It has been documented time and again that a high AEC level could be associated with hematologic malignancies [1, 4, 11, 25, 30]. Furthermore, eosinophilia patients visiting premium checkup clinics had a significantly higher likelihood of being screened for potential causations and interventions than those visiting GP clinics. As premium checkup clinics are mainly serviced by family medicine staff who have more experience than interns in GP clinics, we speculate that the former have more knowledge regarding eosinophilia and thus were more equipped to detect and diagnose this condition.

To our knowledge, this is one of the few studies in the literature on the prevalence of eosinophilia detected in periodic health examinations and the subsequent management by physicians. Additionally, the follow-up period to determine the outcome of eosinophilia patients in this study was sufficiently long to yield a reasonable conclusion concerning the nature and extent of the problem and possible solutions. However, our study had some limitations. First, the retrospective study design resulted in the loss of some information, such as the uncertainty regarding the physician’s management (medical history, physical examination, laboratory investigation, and anthelmintic drug therapy). We assume that when physicians entertained the diagnosis of eosinophilia, they intended to identify its cause. For example, if we observed a physician’s diagnosis of eosinophilia, we concluded that the physician conducted an examination for hepatosplenomegaly or lymphadenopathy as the possible cause of eosinophilia. However, some physicians routinely conduct examinations for hepatosplenomegaly or lymphadenopathy during periodic health checkups, regardless of whether they were seeking to identify the cause of eosinophilia. Second, we could not definitively conclude whether “spontaneous recovery” indicated full and spontaneous recovery from eosinophilia or whether specific treatment from other health care providers was received, such as in the form of over-the-counter anthelmintic drugs. Third, we could not assess the outcome in one-third of our patients because of insufficient data from a one-time visit. Finally, this study was conducted in single tertiary hospital; thus, our results might not be representative of the general population.

We suggest that physicians should recognize the importance and high prevalence of eosinophilia because eosinophilia can be an indicative symptom of serious diseases, such as malignancy. Thus, a complete medical history and physical examination should be conducted for all patients once a diagnosis of eosinophilia has been established, and further laboratory investigation may be performed based on the possible causations whose involvement is suspected. In endemic areas where parasite infections are common, in addition to stool examination, serology tests for common tissue-invasive parasites should be considered. Empirical anthelmintic drug therapy is another option in developing countries and the regimen depends on epidemiological studies in each area. Follow-up CBC is also important in the management of eosinophilia patients. Follow-up CBC should be performed until a resolution is achieved or until the cause of eosinophilia is identified. In patients with persistent eosinophilia, invasive tests should be considered to determine possible serious underlying causes, such as malignancies. Additionally, efforts should be made to create awareness and increase knowledge among physicians regarding eosinophilia, particularly in newly graduated doctors. Prospective cohort studies may be conducted in the future to confirm the ideal course of management in eosinophilia patients, such as the need for follow-up CBC after anthelmintic drug therapy to confirm the presence of parasite infections. Future multi-center studies may contribute new insights into the investigation and treatment of eosinophilia.

## Conclusion

Eosinophilia is a common abnormal condition frequently detected in periodic health examinations. Most general physicians have limited knowledge on its diagnosis and underlying cause, especially in patients with mild severity. Empirical anthelmintic therapy showed satisfactory results for the management of eosinophilia in endemic areas prone to parasite infections. However, further investigation is required in patients with persistent eosinophilia to identify the underlying cause, especially in those with malignancies.
